# Graph kernels for chemoinformatics – a critical discussion

**DOI:** 10.1186/1758-2946-3-S1-O8

**Published:** 2011-04-19

**Authors:** Matthias Rupp

**Affiliations:** 1 Helmholtz Zentrum München, Deutsches Forschungszentrum für Umwelt & Gesundheit, Ingolstädter Landstr. 1, 85764 Neuherberg, Germany

## 

We analyze the use, advantages, and drawbacks of graph kernels in chemoin-formatics, including a comparison of kernel-based approaches with other methodology, as well as examples of applications.

Kernel-based machine learning [1], now widely applied in chemoinformatics, delivers state-of-the-art performance [2] in tasks like classification and regression. Molecular graph kernels [3] are a recent development where kernels are defined directly on the molecular structure graph. This allows the adaptation of methods from graph theory to structure graphs and their direct use with kernel learning algorithms. The main advantage of kernel learning, the so-called "kernel trick", allows for a systematic, computationally feasible, and often globally optimal search for non-linear patterns, as well as the direct use of non-numerical inputs such as strings and graphs. A drawback is that solutions are expressed indirectly in terms of similarity to training samples, and runtimes that are typically quadratic or cubic in the number of training samples.

Graph kernels [3] are positive semidefinite functions defined directly on graphs. The most important types are based on random walks, subgraph patterns, optimal assignments, and graphlets. Molecular structure graphs have strong properties that can be exploited [4], e.g., they are undirected, have no self-loops and no multiple edges, are connected (except for salts), annotated, often planar in the graph-theoretic sense, and their vertex degree is bounded by a small constant. In many applications, they are small. Many graph kernels are general-purpose, some are suitable for structure graphs, and a few have been explicitly designed for them.

We present three exemplary applications of the iterative similarity optimal assignment kernel [5], which was designed for the comparison of small structure graphs: The discovery of novel agonists of the peroxisome proliferator-activated receptor γ [6] (ligand-based virtual screening), the estimation of acid dissociation constants [7] (quantitative structure-property relationships), and molecular de novo design [8].
